# Joint contributions of psychological distress and demanding working conditions to short and long sickness absence among young and early midlife municipal employees

**DOI:** 10.1093/eurpub/ckaf048

**Published:** 2025-04-08

**Authors:** Anna C Svärd, Mari-Liis Kalima, Jaana I Halonen, Minna Mänty, Tero Kujanpää, Eira Roos, Jatta Salmela, Tea Lallukka

**Affiliations:** Department of Public Health, Faculty of Medicine, University of Helsinki, Helsinki, Finland; Department of Public Health, Faculty of Medicine, University of Helsinki, Helsinki, Finland; Department of Public Health, Finnish Institute for Health and Welfare (THL), Helsinki, Finland; Division of Psychobiology and Epidemiology, Department of Psychology, Stockholm University, Stockholm, Sweden; Department of Public Health, Faculty of Medicine, University of Helsinki, Helsinki, Finland; Department of Strategy and Research, City of Vantaa, Vantaa, Finland; Research Unit, Social Insurance Institution of Finland, Helsinki, Finland; Department of Public Health, Faculty of Medicine, University of Helsinki, Helsinki, Finland; Department of Public Health, Faculty of Medicine, University of Helsinki, Helsinki, Finland; Department of Public Health, Faculty of Medicine, University of Helsinki, Helsinki, Finland

## Abstract

This register-linked follow-up study examined whether psychological distress and demanding working conditions are jointly associated with short and long sickness absence (SA) periods among young and midlife Finnish public sector employees. We linked the Helsinki Health Study survey (response rate 51.5%, 80% women, ages 19–39 years in 2017) on psychological distress, physically and mentally strenuous work, and hours per day spent in physical work with the employer’s SA register (*n* = 3609, mean follow-up of 2.1 years). We calculated rate ratios (RRs) and their 95% confidence intervals (CIs) for short (1–7 days) and long (8+ days) SA periods using negative binomial regression models. Additionally, we calculated the synergistic interaction between psychological distress and working conditions. Most (88%) participants had at least one short and 31% at least one long SA period. Participants with psychological distress and exposure to demanding working conditions had the highest RRs for long SA periods (physically strenuous work: RR: 2.27, CI: 1.87–2.77; mentally strenuous work: RR: 2.02, CI: 1.66–2.46; ≥3 h per day spent in physical work: RR: 2.41, CI: 1.94–2.99). The interactions for long SA were negative for physically demanding working conditions, but additive for mentally strenuous work. The associations were weaker for short SA periods. Adjusting for other covariates only slightly attenuated these associations. Psychological distress and demanding working conditions were jointly associated with short and long SA periods. Both individual- and workplace-related risk factors for SA need to be considered when planning preventive actions.

## Introduction

Mental health is a rising public health concern [1]; symptoms of depression and anxiety have increased in most OECD countries in recent years [2], and mental disorders are a major cause of sickness absence (SA) in Northern Europe [3–5]. Also, non-specific symptoms of mental ill-health, such as psychological distress, are associated with SA [6, 7]. In Finland, especially young employees’ mental health is a concern, as the prevalence of psychological distress doubled between 2017 and 2022 [8, 9], and the increase in SA due to mental disorders has been sharp among young and early midlife adults [3, 4].

There are several phenomena associated with the increase seen in mental ill-health, including work-related factors [2–4, 10]. Studies have shown that exposure to demanding physical and mental working conditions is associated with SA [11–15]. Mental strenuousness of work has increased, especially among women and younger age groups, during the last years, whereas physical strenuousness of work has slightly reduced in Western countries [16, 17]. However, especially those with low socioeconomic positions still report their job as physically demanding [16].

Even though both psychological distress and demanding working conditions are associated with SA, there are few previous studies, which have examined their joint association with SA [18–20]. A review focusing on prognostic factors for SA among employees with common mental disorders included only work-related psychosocial factors and focused mainly on long SA periods [19]. A Danish study showed a dose–response association between demanding physical working conditions and long SA periods; however, psychosocial working conditions did not interact with physical working conditions in long SA periods [20]. We are aware of only one previous study, which examined physical working conditions and short SA periods [18]. The study showed that the association with SA was the strongest for midlife and aging Finnish employees with common mental disorders and exposure to physical workload or hazardous exposure simultaneously, the associations being stronger the longer the SA period was. Short SA periods are common among young employees [21] and are associated with long-term and permanent work disability [22] and are therefore important to study. Understanding the interactions between psychological distress and workplace-related factors can be useful when planning early intervention points. A recent study from the UK showed that especially preventing mental disorders is important in reducing SA and would deliver benefits to the economy [23]. Early interventions could guarantee that the decreasing pool of the workforce in aging societies maintains a high level of workability.

This register-linked follow-up study examined the joint association of psychological distress, and exposure to demanding working conditions with subsequent short and long SA periods among young and early midlife employees. We calculated the synergistic interaction between psychological distress and working conditions using the synergy index (S), which is calculated from the additive rates of the dichotomized exposures [24]. An additional aim was to study the potential contribution of age, gender, marital status, education, alcohol use, smoking, leisure-time physical activity (LTPA), and body mass index (BMI) to the association—factors which in previous studies have been associated with working conditions, psychological distress, or SA [19, 25, 26].

## Methods

### Participants

**Figure 1.**
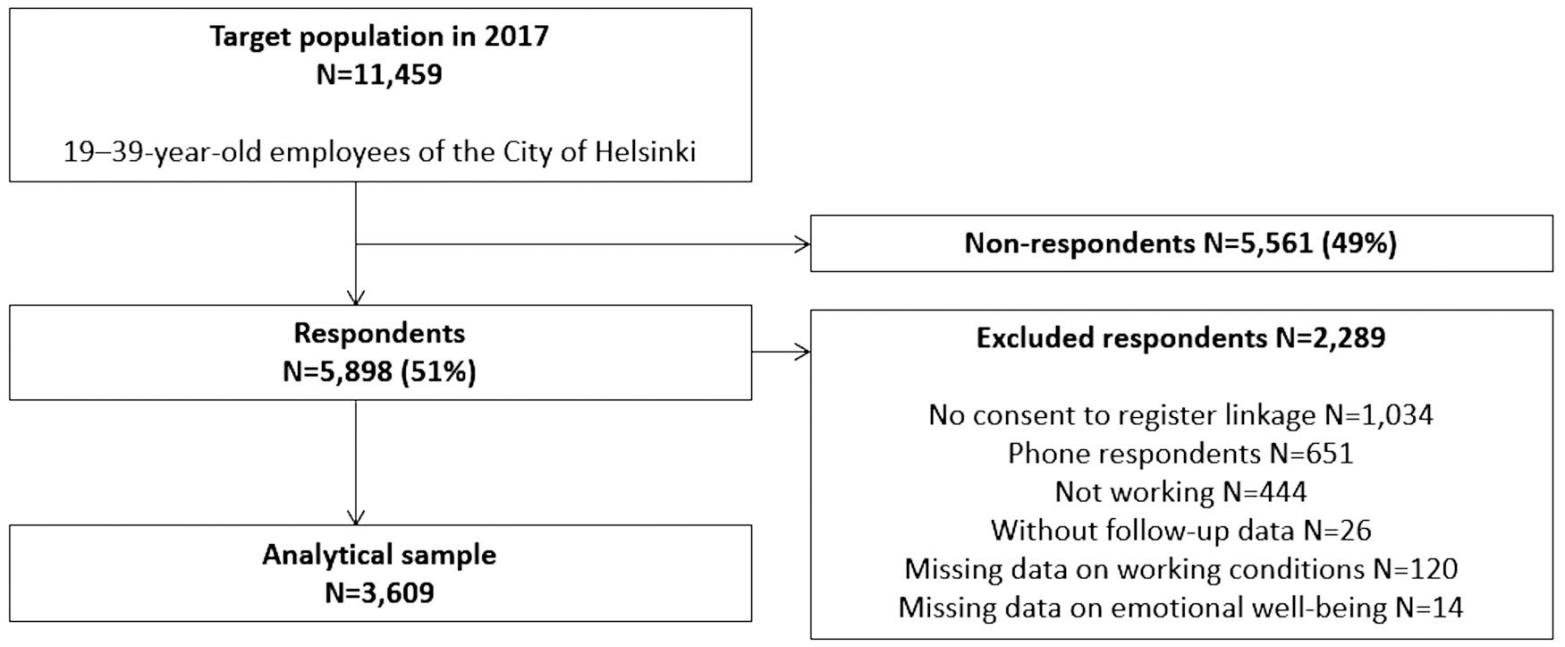
Flow chart of study population selection.

The Helsinki Health Study is an ongoing cohort study on employees of the City of Helsinki, Finland [27]. The City of Helsinki has around 38 000 employees representing a wide range of occupations and sectors. Data were collected in 2017 via online and postal questionnaires among 19–39-year-old employees with at least a 50% employment contract for a minimum of 4 months before the start of the data collection (*n* = 11 459) (Fig. 1). Additionally, a shorter telephone interview (*n* = 787) was conducted among those who did not respond to the online or postal questionnaire. In total, 5898 participants responded (response rate 51.5%), and of these, 4864 (82%) consented to register linkage. Most respondents were women (78%), which reflects the gender distribution of the target population and the municipal sector in Finland [27]. According to the non-response analysis, the respondents represented the target population broadly, although those with a lower socioeconomic position and a long SA were both less likely to respond [27].

Of the participants who consented to register linkage, we excluded those interviewed by telephone (*n* = 651) because the interview did not include information on all key variables needed. We also excluded participants who were not working full- or part-time at baseline (*n* = 444), without follow-up data (*n* = 26), and with missing information on emotional well-being (*n* = 14) or working conditions (*n* = 120). The final dataset consisted of 2826 women and 783 men.

### Exposures

#### Working conditions

We used three measures of demanding working conditions, derived from the questionnaire survey. First, single-item questions with four response options ranging from very light to very strenuous were used to measure physical and mental strenuousness of work [28, 29]. Employees with physically very strenuous work were few (*n* = 141), whereas employees with mentally very (*n* = 634) or rather (*n* = 2225) strenuous work were many. Therefore, we dichotomized physically strenuous work into strenuous (rather/very strenuous) and non-strenuous (very/rather light) and mentally strenuous work into strenuous (very strenuous) and non-strenuous (rather/very light and rather strenuous). We also asked how many hours per day the respondents spent doing physically strenuous work, such as heavy lifting and climbing stairs [13, 29], and dichotomized participants into those spending ≥3 h (highest quartile) and <3 h per day in physical work.

#### Psychological distress

We derived information on participant’s psychological distress from the survey, measuring it with the emotional well-being subscale of the RAND-36, which is a widely used measure for assessing health-related quality of life [30, 31]. This subscale, similar to the Mental Health Inventory (MHI-5), consists of five questions reflecting mental well-being during the last 4 weeks, with six response options ranging from “all of the time” to “none of the time”: “Have you been a very nervous person?”, “Have you felt so down in the dumps that nothing could cheer you up?”, “Have you felt calm and peaceful?”, “Have you felt downhearted and blue?”, and “Have you been a happy person?”. The questions were recoded to a value from 0 to 100, and the third and fifth questions were reverse-coded. After that, we calculated an average score ranging from 0 to 100 for those who had answered all questions of the subscale and dichotomized the participants using a cut-off point of 60. In previous studies, 60 points or less have been considered to indicate moderate or severe psychological distress [32, 33]. Additionally, 60 points have been suggested as the best cut-off point for dichotomization in a study focusing on the predictive value of mental health for long-term SA [34] and is optimal for minimizing the misclassification rate [33].

### Outcome measure: sickness absence

We received information on SA from the personnel register of the City of Helsinki for those respondents who gave their consent for register linkage. The follow-up of SA started the day after we received the questionnaire from a participant and continued until March 2020 (the beginning of the COVID-19 pandemic in Finland) or until the end of the participant’s employment contract, whichever came first (mean follow-up of 2.1 years). We examined separately short SA periods of 1–7 days and long SA periods of 8+ days, because a week is the maximum length of self-certified SA periods, which in certain circumstances can be authorized by supervisors without a medical certificate.

### Covariates

Covariates were derived from the survey. We used three age groups: 19–29, 30–34, and 35–39 years. We classified gender as women and men, marital status as married/cohabiting and others (single, divorced, or widowed), and education as upper secondary school or less, bachelor’s degree, and master’s degree or higher. We dichotomized alcohol use into those consuming alcohol weekly and those using alcohol less often than weekly/not at all and smoking status into smokers (current daily and occasional) and non-smokers. BMI was calculated from self-reported weight and height (kg/m^2^), and participants were dichotomized into those with obesity (BMI ≥30 kg/m^2^) and those without obesity (BMI <30 kg/m^2^). We calculated LTPA from the estimates of average weekly hours of LTPA per four intensity grades and multiplied weekly hours by the metabolic equivalent of task (MET) values, then summed the values [35]. We dichotomized participants into those with low and high LTPA using a cut-off of 20 MET-hours [35]. As LTPA might work as a mediator between physical working conditions and SA, we also tested models without LTPA.

Participants who had missing information on marital status (*n* = 8), education (*n* = 4), alcohol use (*n* = 131), smoking (*n* = 5), BMI (*n* = 41), or physical activity (*n* = 47) were classified as single, having low education, non-weekly alcohol users, non-smokers, without obesity, and having high LTPA, respectively, to reduce selection bias, which might be associated with non-response. However, we conducted sensitivity analyses in which we treated participants with missing data as separate subcategories, and the results remained similar.

### Statistical analyses

First, we used cross-tabulation to describe the distribution of characteristics among the participants at baseline according to short and long SA periods. Second, we used negative binomial regression to calculate the incidence and 95% confidence intervals (CIs) for short and long SA periods per 100 person-years by three exposure groups. The exposure groups were formed based on psychological distress and the three measures of demanding working conditions (exposure to physically and mentally strenuous work and hours per day spent in physical work) and categorized them as: participants (1) without psychological distress and without a demanding working condition, (2) without psychological distress and with a demanding working condition, (3) with psychological distress and without a demanding working condition, and (4) with psychological distress and with a demanding working condition.

Third, we calculated rate ratios (RRs) and 95% CIs for subsequent short and long SA periods using negative binomial regression models. Participants without psychological distress and without exposure to demanding working conditions served as the reference group. Model 1 was adjusted for age and gender, and Model 2 additionally for marital status and education. Model 3 was further adjusted for health behaviors (smoking, alcohol use, and LTPA) and BMI. We included a natural logarithm of the follow-up time as an offset variable in the models to account for different follow-up lengths. We also examined the correlations between the variables, which were relatively weak (Supplementary Table S1).

To complement the main analyses, we conducted sensitivity analyses in which we examined the associations between psychological distress and physically and mentally strenuous working conditions both separately and together (Supplementary Table S2). For this, we formed eight joint groups starting with two categories of participants: those with psychological distress and those without. Each of these categories was further divided into four exposure groups with: (1) physically and mentally non-strenuous work, (2) only physically strenuous work, (3) only mentally strenuous work, and (4) both physically and mentally strenuous work.

Finally, we examined the synergistic interaction between psychological distress and working conditions by calculating the synergy index (S) using the following equation: S = (RR participants with psychological distress and with a demanding working condition − 1)/[(RR participants without psychological distress and with a demanding working condition − 1) + (RR participants with psychological distress without a demanding working condition − 1)] [24]. We calculated the synergy index using RRs from Model 1 (adjusted for age and gender) and Model 3 (fully adjusted). An S = 1 suggests no interaction (exact additivity), S > 1 positive interaction, and S < 1 negative interaction [36]. We used IBM SPSS Statistics 27 for the analyses.

### Statement of ethics

The ethics committees of Department of Public Health at the University of Helsinki (initially 30 November 1998, updated 14 February 2017) and the health authorities of the City of Helsinki (initially 5 October 1999, updated 16 April 2017) approved the Helsinki Health Study protocol. The permission to have access to the employer’s personnel register data was obtained from the City of Helsinki. Appropriate ethical aspects have been followed in all phases of the study, according to the Declaration of Helsinki. Participation in the study was voluntary, and full confidentiality was guaranteed. A written informed consent was obtained for participation before taking part in this study. Additionally, a separate written informed consent was asked for to link the Helsinki Health Study survey to register data.

### Use of AI

We used ChatGPT by OpenAI as a tool for proofreading and editing the language of the article.

## Results

During the 2.1 years of mean follow-up, most participants (88%) had at least one short SA period, and almost one-third (31%) had at least one long SA period (Table 1). Overall, 24% reported psychological distress, 33% physically and 18% mentally strenuous work, and 25% spending ≥3 h per day in physical work.

**Table 1.** Characteristics of the young and midlife employees of the City of Helsinki at baseline in 2017 by subsequent sickness absence (SA) periods of 1–7 days and 8+ days during a mean follow-up of 2.1 years

		SA period of 1–7 days			SA period of 8+ days		
Characteristics at baseline	All *n* (%)	No *n* (%)	Yes *n* (%)	*P*-value^c^	No *n* (%)	Yes *n* (%)	*P*-value^c^
Total *n* (%)	3609 (100)	451 (12)	3158 (88)		2498 (69)	1111 (31)	
Gender				<.001<0.001			<.001
Women	2826 (78)	313 (11)	2513 (89)		1915 (68)	911 (32)	
Men	783 (22)	138 (18)	645 (82)		583 (74)	200 (26)	
Age (years)				.060			.440
18–29	1162 (32)	138 (12)	1024 (88)		788 (68)	374 (32)	
30–34	1186 (33)	170 (14)	1016 (86)		826 (70)	360 (30)	
35–39	1261 (35)	143 (11)	1118 (89)		884 (70)	377 (30)	
Marital status				.728			.651
Others	1254 (35)	160 (13)	1094 (87)		862 (69)	392 (31)	
Married/cohabiting	2355 (65)	291 (12)	2064 (88)		1636 (69)	719 (31)	
Education				<.001			<.001
Upper secondary school or less	1189 (33)	106 (9)	1083 (91)		737 (62)	452 (38)	
Bachelor’s degree	1324 (37)	160 (12)	1164 (88)		897 (68)	427 (32)	
Master’s degree or higher	1096 (30)	185 (17)	911 (83)		864 (79)	232 (21)	
Alcohol use				<.001			<.001
Weekly	1056 (29)	178 (17)	878 (83)		777 (74)	279 (26)	
Occasional or never	2553 (71)	273 (11)	2280 (89)		1721 (67)	832 (33)	
Smoking				.003			.007
Current	898 (25)	87 (10)	811 (90)		589 (66)	309 (34)	
Former or never-smoker	2711 (75)	364 (13)	2347 (87)		1909 (70)	802 (30)	
Physical activity				.250			.065
Low	554 (15)	61 (11)	493 (89)		365 (66)	189 (34)	
High	3055 (85)	390 (13)	2665 (87)		2133 (70)	922 (30)	
Body mass index^a^				.158			<.001
Living with obesity	536 (15)	57 (11)	479 (98)		308 (57)	228 (43)	
Not living with obesity	3073 (85)	394 (13)	2679 (87)		2190 (71)	883 (29)	
Physically strenuous work				.013			<.001
Strenuous	1177 (33)	124 (11)	1053 (89)		734 (62)	443 (38)	
Non-strenuous	2432 (67)	327 (13)	2105 (87)		1764 (73)	668 (27)	
Mentally strenuous work				.060			<.001
Strenuous	634 (18)	65 (10)	569 (90)		394 (62)	240 (38)	
Non-strenuous	2975 (82)	386 (13)	2589 (87)		2104 (71)	871 (29)	
Time spent in physical work				.012			<.001
≥3 h/workday	883 (24)	89 (10)	794 (90)		530 (60)	353 (40)	
<3 h/workday	2726 (76)	362 (13)	2364 (87)		1968 (72)	758 (28)	
Psychological distress^b^				.074			<.001
Moderate	857 (24)	92 (11)	765 (89)		517 (60)	340 (40)	
No	2752 (76)	359 (13)	2393 (87)		1981 (72)	771 (28)	

a Body mass index: not living with obesity (<30 kg/m^2^) and living with obesity (≥30 kg/m^2^).

b Emotional well-being score: moderate psychological distress ≤60 points, no psychological distress >60 points.

c *P*-values from chi-squared tests.

We examined whether the associations between the exposure groups and SA differed between women and men and found no significant gender interactions for physically strenuous work (*P* = .724 for short and *P* = .745 for long SA periods), mentally strenuous work (*P* = .629 for short and *P* = .961 for long SA periods), or hours per day spent in physical work (*P* = .876 for short and *P* = .645 for long SA periods).

**Figure 2.**
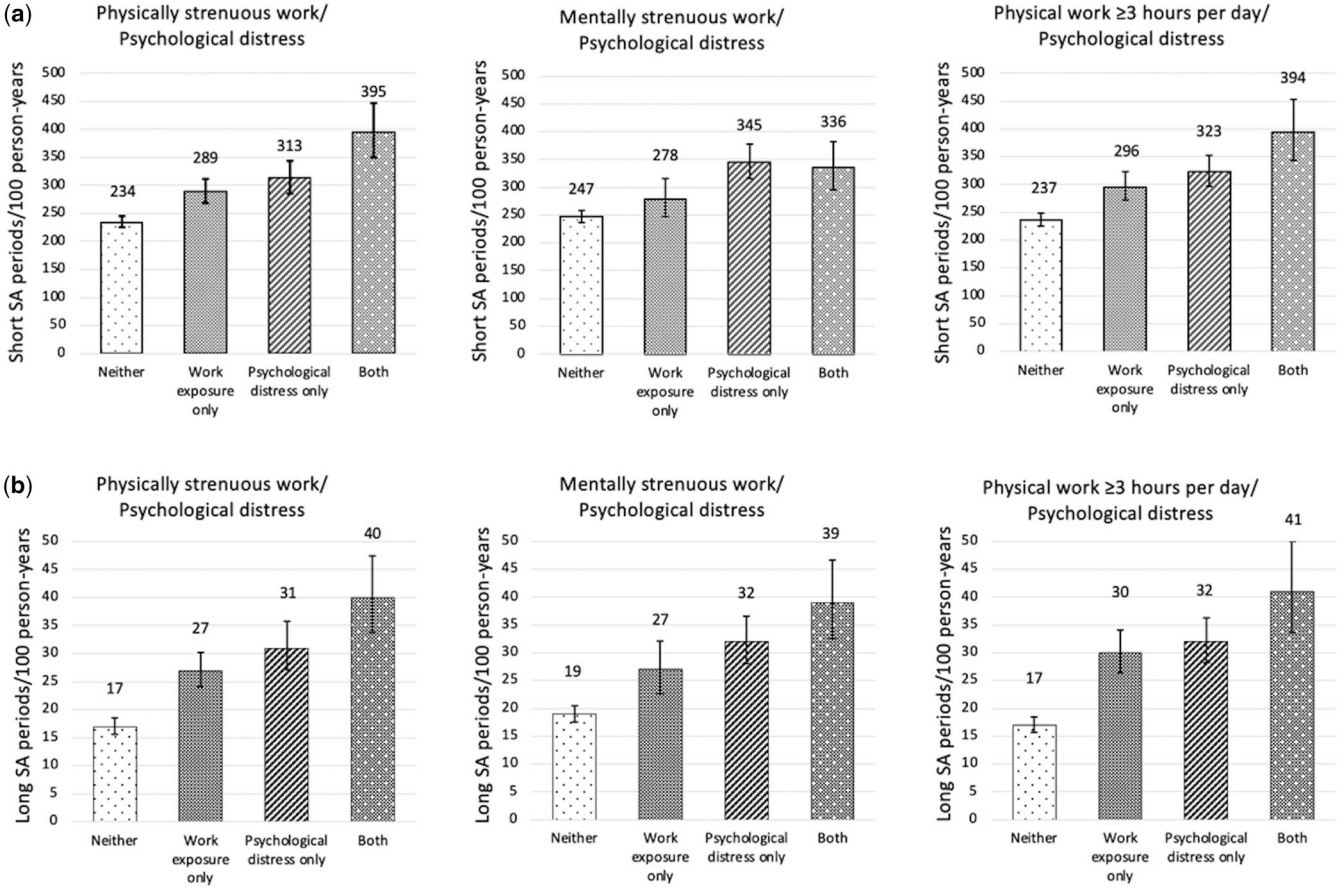
Age- and gender-adjusted incidence of sickness absence (SA) periods with 95% confidence intervals of (a) short 1–7 days and (b) long 8+ days per 100 person-years for participants with psychological distress and exposure to demanding working conditions (young and midlife Helsinki health study participants, *n* = 3609, from 2017).

Figure 2 shows that participants with psychological distress and exposure to demanding working conditions had the highest age- and gender-adjusted incidence of long SA periods per 100 person-years. Participants with psychological distress and exposure to physically demanding working conditions also had the highest incidence of short SA periods, whereas for mentally strenuous work, the incidence was similar for participants with both exposures and for participants with psychological distress only. Additionally, participants with psychological distress or exposure to a demanding working condition seemed to have a higher incidence of SA periods than participants with neither exposure.

Table 2 presents RRs for short and long SA periods by exposure groups. Participants with psychological distress and exposure to demanding working conditions had the highest RRs for long SA periods (physically strenuous work: RR: 2.27, CI: 1.87–2.77; mentally strenuous work: RR: 2.02, CI: 1.66–2.46; ≥3 h per day spent in physical work: RR: 2.41, CI: 1.94–2.99). For short SA periods, associations were similar but weaker for physically strenuous work and hours per day spent in physical work. However, for mentally strenuous work, RRs were similar for participants with psychological distress and exposure to mentally strenuous work (RR: 1.33, CI: 1.16–1.53) and participants with psychological distress only (RR: 1.37, CI: 1.24–1.52). Adjustment for Model 2 attenuated somewhat the associations, especially for physical working conditions and short SA periods, for which the associations lost statistical significance. Other adjustments had minimal effects on the associations.

**Table 2.** Rate ratios (RRs) and their 95% confidence intervals (95% CIs) for sickness absence (SA) periods of 1–7 days and 8+ days by young and midlife employees of the City of Helsinki with and without moderate psychological distress (emotional well-being score ≤60), and with and without exposure to demanding working conditions in 2017

			Model 1			Model 2			Model 3		
SA periods	Exposure group	*n* (%)	RR	95% CI	S	RR	95% CI	S	RR	95% CI	S
1–7 days	Physically strenuous work/psychological distress				1.05			1.25			1.19
	Neither	1883 (52)	1.00			1.00			1.00		
	Work exposure only	869 (24)	1.19	1.09–1.31		1.01	0.92–1.11		1.01	0.92–1.11	
	Psychological distress only	549 (15)	1.33	1.19–1.47		1.27	1.14–1.41		1.25	1.12–1.39	
	Both	308 (9)	1.60	1.40–1.83		1.35	1.18–1.55		1.31	1.15–1.51	
	Mentally strenuous work/psychological distress				0.66			0.72			0.70
	Neither	2391 (66)	1.00			1.00			1.00		
	Work exposure only	361 (10)	1.13	1.00–1.27		1.15	1.01–1.30		1.15	1.02–1.30	
	Psychological distress only	584 (16)	1.37	1.24–1.52		1.31	1.19–1.45		1.28	1.16–1.42	
	Both	273 (8)	1.33	1.16–1.53		1.33	1.16–1.53		1.30	1.14–1.50	
	Physical work ≥3 h per day/psychological distress				1.02			1.00			1.00
	Neither	2102 (58)	1.00			1.00			1.00		
	Work exposure only	650 (18)	1.24	1.12–1.36		1.05	0.95–1.16		1.05	0.94–1.16	
	Psychological distress only	624 (17)	1.35	1.22–1.49		1.30	1.17–1.43		1.27	1.15–1.40	
	Both	233 (6)	1.60	1.38–1.86		1.35	1.15–1.57		1.32	1.13–1.54	
8+ days	Physically strenuous work/psychological distress				0.86			0.73			0.71
	Neither	1883 (52)	1.00			1.00			1.00		
	Work exposure only	869 (24)	1.60	1.39–1.85		1.29	1.11–1.51		1.29	1.10–1.50	
	Psychological distress only	549 (15)	1.88	1.60–2.21		1.83	1.55–2.15		1.78	1.50–2.10	
	Both	308 (9)	2.27	1.87–2.77		1.82	1.48–2.23		1.76	1.44–2.17	
	Mentally strenuous work/psychological distress				0.92			0.96			0.92
	Neither	2391 (66)	1.00			1.00			1.00		
	Work exposure only	361 (10)	1.43	1.18–1.73		1.44	1.19–1.74		1.45	1.19–1.76	
	Psychological distress only	584 (16)	1.58	1.44–1.95		1.61	1.38–1.88		1.58	1.35–1.85	
	Both	273 (8)	2.02	1.66–2.46		2.01	1.65–2.46		1.95	1.59–2.38	
	Physical work ≥3 h per day/psychological distress				0.83			0.73			0.73
	Neither	2102 (58)	1.00			1.00			1.00		
	Work exposure only	650 (18)	1.82	1.56–2.12		1.49	1.27–1.75		1.47	1.25–1.73	
	Psychological distress only	624 (17)	1.88	1.62–2.19		1.83	1.57–2.13		1.77	1.52–2.07	
	Both	233 (6)	2.41	1.94–2.99		1.96	1.56–2.45		1.91	1.52–2.39	

Synergy index (S) is the interaction of psychological distress and exposure to demanding working conditions to SA.

Model 1: adjusted for age and gender.

Model 2: adjusted for Model 1 + marital status and education.

Model 3: adjusted for Model 2 + alcohol use, smoking, leisure-time physical activity, and body mass index.

The interaction of psychological distress and physically strenuous work (Model 1: S = 0.86; Model 3: S = 0.71) and hours per day spent in physical work (Model 1: S = 0.83; Model 3: S = 0.73) was negative for long SA periods (Table 2). For short SA periods, the interaction was positive for physically strenuous work (Model 1: S = 1.15, Model 3: S = 1.19) and additive for ≥3 h per day spent in physical work (Model 1: S = 1.02; Model 3: S = 1.00). For mentally strenuous work, the interaction was negative for short (Model 1: S = 0.66; Model 3: S = 0.70) and additive (Model 1: S = 1.01) or weakly negative (Model 3: S = 0.92) for long SA periods.

Supplementary Table S2 shows similar results for physically and mentally strenuous work, separately and combined. However, RRs for psychological distress and physically strenuous work remained consistent, while the highest RRs for long SA periods occurred when both working conditions were present.

## Discussion

Our study showed that participants with both psychological distress and exposure to physically or mentally demanding working conditions had the strongest association with SA periods of 8+ days. Both psychological distress and demanding working conditions were also independently associated with long SA periods. For long SA periods, interactions were negative for physically strenuous work and hours per day spent in physical work, meaning their combined impact with psychological distress is less than the sum of the exposures individually. For mentally strenuous work, the interaction was additive, indicating no mutual amplification of the association. This is encouraging, as many employees face multiple exposures. For short SA periods, the associations were weaker: participants with both psychological distress and exposure to demanding physical working conditions had the strongest association with SA, but adjustment for education attenuated the associations. The interactions were weakly positive for physically strenuous work and additive for hours per day spent in physical work. The associations with short SA periods were similar among participants with psychological distress, regardless of mental strenuousness of work, and the interaction was negative.

Sensitivity analyses showed the strongest association with long SA periods in participants with psychological distress and both work strains, while mentally strenuous work did not further increase the association for short SA. In our study, the interaction between psychological distress and physical working conditions was additive/weakly positive for short SA periods, however, negative for long SA periods, meaning that one exposure was not additive to the other, even when using a higher cut-off of 15+ SA days (results not shown). This might be because long SA periods among younger employees are most often caused by mental disorders, whereas musculoskeletal diseases, which are more strongly related to physical working conditions, are the major cause of SA among older employees [4]. It would be important for further studies to examine SA diagnoses and elaborate the differences between age groups.

The association with short SA periods was similar among participants with psychological distress regardless of mental strenuousness of work. Short SA periods, which are common among young employees [21], can be granted without a medical certificate from a physician, for example, in cases of infection. A previous study has shown that there are occupational class differences in the usage of self-certified short SA [37]. Short SA reflects also other than purely health-related issues; it is known that factors, such as the level of compensation during absenteeism, social norms, and employment level, play a role in short SA [38]. It could be that the threshold for SA is higher for employees with psychological distress and mentally demanding work; for example, employees with desktop work can often work from home during an infection, whereas employees with physically demanding work cannot perform their tasks when ill. We adjusted our analyses for education, but it did not attenuate the association between mentally strenuous work and short SA, as it did for physical work. Future studies should aim to deepen the understanding of short SA. By increasing the understanding of short SA, for example, by examining reasons behind them, valuable insight could be gained when aiming to reduce SA.

### Methodological considerations

The data enabled adjustment for several sociodemographic and health-related covariates. However, information on working conditions, emotional well-being, and covariates was self-reported, which may cause over- or underestimation. Psychological distress was assessed with the emotional well-being subscale of RAND-36, a widely used measure of health-related quality of life [30–32]. Emotional well-being measures general mental ill-health symptoms but cannot confirm disorders, aligning with our focus. While participants with psychological distress may rate their jobs as more mentally demanding, the correlation was weak (Supplementary Table S1). The joint contributions could be due to reporting tendencies, which cannot be ruled out in the current design. Despite this, our study shows that having both risk factors differs from reporting only one.

We measured working conditions with single-item measures but also did sensitivity analyses using a multi-item measure including physical workload, hazardous exposure, and computer work [39]. Results were similar for physical workload and hazardous exposure (data not shown). Additionally, a previous study has shown that the single-item measure of mentally strenuous work used in this study correlates with the Karasek’s model of job demands and job strain, but not with job control [29].

Although the non-response analysis showed that the respondents broadly represented the target population [27] and the response rate was acceptable, non-response and possible selection of healthy workers remains a limitation. Additionally, most participants were women, which reflects the gender distribution of the target population and municipal sector in Finland [27]. Because the study included only Finnish public sector employees, the generalizability of the results may be limited.

A major strength of this study is the use of a large employee cohort, including young and midlife employees, representing a wide range of different occupations. Another strength is the use of the employer’s register data on SA periods and the longitudinal design. The data included all SA periods, also short SA periods, which are covered by the employer and can be self-certified for 1–7 days. This is a strength because short SA periods are less studied, as they are not included in national registers. However, short SA periods are common, especially among the young employees [21], and are known to predict longer SA periods [22]. Therefore, information on them can be important for planning early interventions.

## Supplementary Material

ckaf048_Supplementary_Data

## Data Availability

Data sharing statement The Helsinki Health Study survey data cannot be made publicly available due to strict data protection laws and regulations. The data can only be used for scientific research. More information on the survey data can be requested from the Helsinki Health Study research group (kttl-hhs@helsinki.fi). Key points Among young and early midlife Finnish employees, psychological distress and exposure to demanding working conditions were individually associated with SA. The strongest associations were found among participants reporting both exposures simultaneously. Both individual- and workplace-related risk factors for SA are important to be considered when planning preventive actions; for example, by identifying participants with psychological distress and demanding working conditions and supporting their work ability and by offering targeted support and modifying their working conditions.
