# Supplementary material for: Joint contributions of psychological distress and demanding working conditions to short and long sickness absence among young and early midlife municipal employees
Source: Eur J Public Health. 2025 Apr 8;35(3):534–40. doi: 10.1093/eurpub/ckaf048 (PMC12192422; doi:10.1093/eurpub/ckaf048)
Supplement: ckaf048_Supplementary_Data [file ckaf048_supplementary_data.docx]

Supplemental Table S1. Rate ratios (RRs) and their 95% confidence intervals (95% CIs) for sickness absence (SA) periods of 1–7 days and 8+ days by young and midlife Helsinki Health Study participants (n=3,609) with and without moderate psychological distress (emotional well-being score ≤60), and with and without exposure to demanding working conditions (multi–item measure: physical workload, hazardous exposure, and computer work) in 2017.

| SA periods |  | |  | | Model 1 | | | | Model 2 | | | | | | Model 3 | | | | |  |
| --- | --- | --- | --- | --- | --- | --- | --- | --- | --- | --- | --- | --- | --- | --- | --- | --- | --- | --- | --- | --- |
|  | Exposure group | | n (%) | | RR | | 95% CI | | RR | | | 95% CI | | | RR | | | 95% CI | |  |
| 1–7 days | Physical workload/psychological distress | |  | |  | |  | |  | | |  | | |  | | |  | |  |
|  | Neither | | 2148 (60) | | 1.00 | |  | | 1.00 | | |  | | | 1.00 | | |  | |  |
|  | Work exposure only | | 604 (17) | | 1.39 | | 1.26–1.53 | | 1.22 | | | 1.10–1.35 | | | 1.21 | | | 1.09–1.34 | |  |
|  | Psychological distress only | | 612 (17) | | 1.35 | | 1.22–1.49 | | 1.32 | | | 1.19–1.46 | | | 1.29 | | | 1.16–1.43 | |  |
|  | Both | | 245 (7) | | 1.72 | | 1.49–1.99 | | 1.46 | | | 1.26–1.70 | | | 1.43 | | | 1.23–1.66 | |  |
|  | Hazardous exposures/psychological distress | |  | |  | |  | |  | | |  | | |  | | |  | |  |
|  | Neither | | 2094 (58) | | 1.00 | |  | | 1.00 | | |  | | | 1.00 | | |  | |  |
|  | Work exposure only | | 658 (18) | | 1.26 | | 1.14–1.38 | | 1.20 | | | 1.09–1.33 | | | 1.20 | | | 1.09–1.33 | |  |
|  | Psychological distress only | | 602 (17) | | 1.36 | | 1.23–1.50 | | 1.32 | | | 1.19–1.46 | | | 1.29 | | | 1.16–1.43 | |  |
|  | Both | | 255 (7) | | 1.57 | | 1.36–1.81 | | 1.45 | | | 1.25–1.67 | | | 1.42 | | | 1.23–1.64 | |  |
|  | Computer work/psychological distress | |  | |  | |  | |  | | |  | | |  | | |  | |  |
|  | Neither | | 2079 (58) | | 1.00 | |  | | 1.00 | | |  | | | 1.00 | | |  | |  |
|  | Work exposure only | | 673 (19) | | 1.03 | | 0.94–1.14 | | 1.14 | | | 1.03–1.26 | | | 1.14 | | | 1.03–1.25 | |  |
|  | Psychological distress only | | 611 (17) | | 1.38 | | 1.25–1.53 | | 1.33 | | | 1.21–1.47 | | | 1.30 | | | 1.18–1.44 | |  |
|  | Both | | 246 (7) | | 1.26 | | 1.09–1.46 | | 1.34 | | | 1.15–1.55 | | | 1.32 | | | 1.13–1.53 | |  |
| 8+ days | Physical workload/psychological distress | |  | |  | |  | |  | | |  | | |  | | |  | |  |
|  | Neither | | 2148 (60) | | 1.00 | |  | | 1.00 | | |  | | | 1.00 | | |  | |  |
|  | Work exposure only | | 604 (17) | | 1.74 | | 1.49–2.03 | | 1.45 | | | 1.23–1.70 | | | 1.44 | | | 1.22–1.69 | |  |
|  | Psychological distress only | | 612 (17) | | 1.87 | | 1.61–2.18 | | 1.85 | | | 1.59–2.17 | | | 1.80 | | | 1.54–2.11 | |  |
|  | Both | | 245 (7) | | 2.21 | | 1.79–2.73 | | 1.78 | | | 1.43–2.21 | | | 1.73 | | | 1.39–2.16 | |  |
|  | Hazardous exposures/psychological distress | |  | |  | |  | |  | | |  | | |  | | |  | |  |
|  | Neither | | 2094 (58) | | 1.00 | |  | | 1.00 | | |  | | | 1.00 | | |  | |  |
|  | Work exposure only | | 658 (18) | | 1.48 | | 1.27–1.72 | | 1.38 | | | 1.18–1.61 | | | 1.38 | | | 1.18–1.61 | |  |
|  | Psychological distress only | | 602 (17) | | 1.76 | | 1.51–2.05 | | 1.70 | | | 1.46–1.99 | | | 1.67 | | | 1.43–1.95 | |  |
|  | Both | | 255 (7) | | 2.20 | | 1.79–2.70 | | 2.03 | | | 1.65–2.50 | | | 1.96 | | | 1.59–2.42 | |  |
|  | Computer work/psychological distress | |  | |  | |  | |  | | |  | | |  | | |  | |  |
|  | Neither | | 2079 (58) | | 1.00 | |  | | 1.00 | | |  | | | 1.00 | | |  | |  |
|  | Work exposure only | | 673 (19) | | 0.96 | | 0.81–1.12 | | 1.10 | | | 0.93–1.30 | | | 1.11 | | | 0.94–1.32 | |  |
|  | Psychological distress only | | 611 (17) | | 1.74 | | 1.50–2.01 | | 1.67 | | | 1.44–1.95 | | | 1.64 | | | 1.41–1.90 | |  |
|  | Both | | 246 (7) | | 1.53 | | 1.22–1.90 | | 1.71 | | | 1.36–2.14 | | | 1.67 | | | 1.33–2.09 | |  |
| *Model 1: Adjusted for age and gender* | |  | |  | |  | |  | | |  | | |  | | |  | | |  |
| *Model 2: Adjusted for Model 1 + marital status and education* | |  | |  | |  | |  | | |  | | |  | | |  | | |  |
| *Model 3: Adjusted for Model 2 + alcohol use, smoking, leisure-time physical activity and body mass index* | | | | | | | | | |  | | |  | | |  | | |  | |

Supplemental Table S2. Rate ratios (RRs) and their 95% confidence intervals (95% CI) for sickness absence (SA) periods of 1–7 days and 8+ days by young and midlife employees of the City of Helsinki with and without moderate psychological distress (emotional wellbeing score ≤ 60) and exposure to physically and/or mentally strenuous work in 2017.

|  | Exposure group | | |  | Model 1 | | | | Model 2 | | | | Model 3 | | | |  |
| --- | --- | --- | --- | --- | --- | --- | --- | --- | --- | --- | --- | --- | --- | --- | --- | --- | --- |
| SA periods | Psychological distress | Physically strenuous work | Mentally strenuous work | n (%) | RR | | 95% CI | | RR | | 95% CI | | RR | 95% CI | | |  |
| 1–7 days |  |  |  |  |  | |  | |  | |  | |  |  | | |  |
|  | – | – | – | 1686 (47) | 1.00 | |  | | 1.00 | |  | | 1.00 |  | | |  |
|  | – | – | + | 197 (5) | 1.08 | | 0.92 –1.27 | | 1.16 | | 0.98–1.37 | | 1.15 | 0.98–1.36 | | |  |
|  | – | + | – | 705 (20) | 1.18 | | 1.07–1.30 | | 1.00 | | 0.90–1.11 | | 1.00 | 0.90–1.11 | | |  |
|  | – | + | + | 164 (5) | 1.31 | | 1.10–1.57 | | 1.13 | | 0.95–1.35 | | 1.14 | 0.96–1.37 | | |  |
|  | + | – | – | 395 (11) | 1.37 | | 1.21–1.54 | | 1.29 | | 1.14–1.46 | | 1.26 | 1.12–1.43 | | |  |
|  | + | – | + | 154 (4) | 1.26 | | 1.05–1.51 | | 1.29 | | 1.07–1.55 | | 1.27 | 1.05–1.53 | | |  |
|  | + | + | – | 189 (5) | 1.63 | | 1.38–1.92 | | 1.36 | | 1.14–1.61 | | 1.32 | 1.11–1.57 | | |  |
|  | + | + | + | 119 (3) | 1.59 | | 1.30–1.95 | | 1.38 | | 1.13–1.69 | | 1.35 | 1.10–1.65 | | |  |
| 8+ days |  |  |  |  |  | |  | |  | |  | |  |  | | |  |
|  | – | – | – | 1686 (47) | 1.00 | |  | | 1.00 | |  | | 1.00 |  | | |  |
|  | – | – | + | 197 (5) | 1.37 | | 1.04–1.79 | | 1.47 | | 1.12–1.93 | | 1.47 | 1.12–1.93 | | |  |
|  | – | + | – | 705 (20) | 1.58 | | 1.34–1.85 | | 1.27 | | 1.07–1.50 | | 1.26 | 1.06–1.49 | | |  |
|  | – | + | + | 164 (5) | 2.02 | | 1.56–2.63 | | 1.65 | | 1.26–2.16 | | 1.66 | 1.27–2.17 | | |  |
|  | + | – | – | 395 (11) | 1.85 | | 1.54–2.24 | | 1.78 | | 1.47–2.15 | | 1.74 | 1.44–2.11 | | |  |
|  | + | – | + | 154 (4) | 2.20 | | 1.68–2.88 | | 2.26 | | 1.72–2.97 | | 2.18 | 1.66–2.88 | | |  |
|  | + | + | – | 189 (5) | 2.21 | | 1.72–2.83 | | 1.72 | | 1.33–2.23 | | 1.68 | 1.30–2.18 | | |  |
|  | + | + | + | 119 (3) | 2.57 | | 1.94–3.40 | | 2.12 | | 1.59–2.83 | | 2.04 | 1.53–2.72 | | |  |
| *Model 1: Adjusted for age and gender* | | | | | |  | |  | |  | |  | | |  |  | |
| *Model 2: Adjusted for Model 1 + marital status and education* | | | | | | | | | |  | |  | | |  |  | |
| *Model 3: Adjusted for Model 2 + alcohol use, smoking, leisure-time physical activity and body mass index* | | | | | | | | | | | | | | |  |  | |
